# Sucralfate enemas reduce the oxidative tissue damage and preserves the contents of E-cadherin and Β-catenin in colonic mucosa without fecal stream

**DOI:** 10.1590/ACB361007

**Published:** 2021-11-29

**Authors:** Daniela Tiemi Sato, Fabio Guilherme Campos, Paulo Gustavo Kotze, Roberta Laís Santos Mendonça, Danilo Toshio Kanno, José Aires Pereira, Carlos Augusto Real Martinez

**Affiliations:** 1 Fellow Master degree. Postgraduate Program in Surgical Sciences – Universidade Estadual de Campinas (UNICAMP) – Campinas (SP), Brazil.; 2 PhD. Associate Professor. Department of Gastroenterology – Faculdade de Medicina – Universidade de São Paulo (FMUSP) – Sao Paulo (SP), Brazil.; 3 PhD. Postgraduate Program in Health Sciences - Pontifícia Universidade Católica do Paraná (PUCPR) – Curitiba (PR), Brazil.; 4 Fellow Master degree. Postgraduate Program in Health Sciences – Universidade São Francisco (USF) - Bragança Paulista (SP), Brazil.; 5 Fellow PhD degree. Postgraduate Program in Health Sciences - Universidade São Francisco (USF) - Bragança Paulista (SP), Brazil.; 6 PhD. Assistant Professor. Division of Pathology - Faculty of Medicine – Universidade São Francisco (USF) - Bragança Paulista (SP), Brazil.; 7 PhD, Associate Professor. Postgraduate Program in Health Sciences - Universidade São Francisco (USF) - Bragança Paulista (SP). Postgraduate Program in Sciences of Surgery - Universidade Estadual de Campinas (UNICAMP) – Campinas (SP), Brazil.; Universidade Estadual de Campinas, Postgraduate Program in Sciences of Surgery, Campinas, SP, Brazil

**Keywords:** Colitis, Colostomy, Fatty Acids, Volatile, Sucralfate, Cadherins, Catenins, Lipid Peroxidation, Rats

## Abstract

**Purpose:**

To evaluate the effects of sucralfate enemas in tissue contents of E-cadherin and ?-catenin in an experimental diversion colitis.

**Methods:**

Thirty-six male Wistar rats were submitted to a proximal colostomy and a distal mucous fistula. They were allocated into three groups: first group received daily saline enemas (2 mL/day) and the two other groups daily enemas with sucralfate at dosage of 1 or 2 g/kg/day, respectively. Six animals of each group were euthanized after two weeks and six animals after four weeks. The inflammation of the excluded mucosa was evaluated by histological analysis. The oxidative damage was quantified by measurement of malondialdehyde tissue levels. The expression of E-cadherin and ?-catenin was identified by immunohistochemistry, and its contents were quantified by computer-assisted image analysis.

**Results:**

Sucralfate enemas reduced inflammation in animals subjected to treatment with 2 g/kg/day by four weeks, and the levels of oxidative damage in mucosa without fecal stream irrespective of concentration and time of intervention. E-cadherin and ?-catenin content increased in segments without fecal stream in those animals subjected to treatment with sucralfate.

**Conclusions:**

Sucralfate reduces the inflammation and oxidative stress and increases the tissue content of E-cadherin and ?-catenin in colonic mucosa devoid to the fecal stream.

## Introduction

The integrity of the colonic epithelium is essential for the maintenance of human life^1^. The epithelium of the intestinal tract serves as a protective barrier separating luminal contents from underlying tissue compartments^2^. Disruption of epithelial barrier integrity is a significant factor associated with the development of several diseases, including gastroenteritis caused by viruses or bacteria, necrotizing enterocolitis, inflammatory bowel disease (IBD), and diversion colitis (DC)^2^^-^ 5. The mechanism of defense of the epithelial barrier is formed by a layer of specialized and polarized epithelial cells, a mucus tier that covers the intestinal mucosa, intercellular junction systems formed by tight junctions (TJs), adherens junctions (AJs), gap junctions, desmosomes, and a basal membrane. Immune cells, immunoglobulins, and cytokines also play a role in defense of the intestinal mucosa against pathogen infiltration^2^^,^ 5.

DC is an inflammatory process that occurs in colon or rectal mucosa devoid of the intestinal transit^6^. It has been recognized as an energy-deficiency syndrome caused by a lack of short-chain fatty acids (SCFAs) supply to colonic epithelial cells^6^^,^ 7. Under normal conditions, anaerobic bacteria present inside the colonic lumen ferment carbohydrates to produce SCFAs^7^. The energy required by colonic epithelial cells depends on the luminal availability of SCFAs, which mainly include butyrate, propionate, and acetate^8^. Cells of the colonic mucosa require SCFAs to maintain their energetic metabolism and protein synthesis^7^. When there is deficiency of SCFAs, the cells of the colonic mucosa present alterations in their redox state with a decrease in cellular oxidative phosphorylation, resulting in overproduction of reactive oxygen species (ROS), which causes oxidative damage to all defense systems of the epithelial barrier^5^^,^ 7. Luminal deficiency of SCFAs exacerbates DC, whereas supplying the colonic mucosa that is excluded from the fecal stream with nutritional solutions rich in SCFAs improves the trophism of the epithelium, reduces the levels of oxidative tissue damage, increases the synthesis of several proteins, and protects the colonic mucosa against the inflammatory process^9^^-^ 10.

Studies using DC models showed that ROS could damage the mechanisms of defense that make up the epithelial barrier, particularly the glycoprotein constituents of the mucus layer and the proteins of intercellular junctions^11^^-^ 13. The oxidative damage of the epithelial barrier allows the translocation of antigens and bacteria into inner layers of the colonic wall, triggering inflammatory processes characteristic of DC. ROS can damage the proteins E-cadherin and β-catenin, which are the principal proteins of AJs^13^. To reinforce the importance of oxidative stress in the disruption of these proteins, it has been demonstrated that antioxidant substances can prevent the loss of integrity of these proteins, maintaining the integrity of the colonic epithelial intercellular junctions in experimental models of experimental colitis^14^^-^ 17.

Sucralfate (SCF) molecule is composed of sucrose octasulfate and aluminum hydroxide. SCF molecule meets the inflamed mucosa of the gastrointestinal tract, and it adheres firmly on the surface of the ulcerated epithelium, forming a glycoproteic complex covering the damaged surface^18^. SCF has cytoprotective characteristics, promoting tissue healing, increasing the production of epithelium growing factor (EGF), exhibiting anti-inflammatory properties, and providing crucial antioxidant activity^18^.

Due to all these features, SCF has been used for several years to heal different types of cutaneous lesions, such as surgical wounds and those caused by skin ulcers, mucositis, and mucosal ulceration of the gastrointestinal tract^18^^-^ 22. Enemas with SCF have also been considered a promising tool for the treatment of different types of colitis^14^^,^ 23 ^-^ 25. SCF itself or associated with other drugs has been used to treat radiation proctitis, and ulcerative colitis, with satisfactory results^25^^,^ 26.

Notwithstanding, few studies have analyzed the effects of SCF in experimental models of DC^14^^,^ 27 ^-^ 29. They have showed that the use of SCF clysters to colonic segments without fecal stream reduce the inflammatory process of the intestinal mucosa, increase the tissue contents of different types of mucins, decrease the levels of oxidative tissue damage and the leucocytes infiltrate, and promote the healing of the damage epithelium^27^^-^ 29. However, the antioxidant effects of the applications of clysters with SCF on preserving the content of the principal proteins constituents of the AJs have never been evaluated in colon mucosa without fecal transit. Thus, this study aimed to evaluate the antioxidants effects of enemas containing SCF on tissue content of E-cadherin and β-catenin in an experimental model of DC.

## Methods

 This study was performed in accordance with Brazilian Federal Law No. 11,794 and the recommendations of the Brazilian College for Animal Experimentation (COBEA). This study also obeyed the National Institutes of Health Guide for the Care and Use of Laboratory Animals (NIH Publications No. 8,023, revised 1978). This experimental study was approved by the University Research Ethics Committee (Reference number 2,211/07).

### Surgical technique: diversion of the fecal stream

To induce DC, all animals were anesthetized by intraperitoneal administration of 0.1 mL/100 g of 1:1 (v/v) ketamine, at a dosage of 50 mg/mL, and xylazine, at a dosage of 20 mg/mL. The peritoneal cavity was accessed by a midline incision. The left colon was identified, and the vessels of the marginal arcade were tied. Then, the left colon was sectioned, and the cranial segment was exteriorized as a terminal colostomy. The caudal segment of the remaining colon was catheterized and irrigated with saline to completely remove fecal residues remaining in the colic segment to be excluded from intestinal transit. After cleaned, the catheter was removed, and this caudal segment of the colon was exteriorized like a mucous fistula. The surgical wound was closed in two layers of running suture.

After surgical procedure, the animals were isolated in individual cages under controlled temperature and humidity conditions, with 12-hour light-dark cycles. Analgesia was supplied by dipyrone (15 mg/kg) for five consecutive days. There was no particular care for the stomas or abdominal incisions, and antibiotics were not used. After surgery, all animals were kept in individual cages for two weeks to enable the development of DC.

### Experimental groups

Thirty-six *Rattus norvegicus albinus* were divided into three experimental groups with 12 rats in each group. The treatment with the proposed solutions was initiated two weeks after the surgical procedure. The first group of 12 animals received daily saline enemas. The remaining two groups received daily SCF clysters at two different dosages: 1 and 2 g/kg/day. Six rats were euthanized in each experimental group after two weeks of treatment, and the remaining ones were euthanized after four weeks of treatment.

### Sample collection

After finishing the treatment periods (two or four weeks), the animals were anesthetized as previously described, and the previous incision was opened again. Colonic fragments were collected from the colon without fecal stream and subjected to irrigation with saline or SCF at both concentrations.

To standardize the histological analyses, in all animals, the colonic segments without fecal transit were removed 0.5 cm above Peyer’s lymphoid plaque as a standard technique. An additional segment with 1 cm of the colon’s length not exposed to the fecal stream was removed for malondialdehyde (MDA) biochemical analysis. The removed specimens were opened opposite to the mesenteric attachment, washed with phosphate buffered saline (PBS), put in a 10%-buffer formaldehyde, and forwarded to histological and immunohistochemical study. The segments used for MDA quantification were washed in saline, placed in an Eppendorf cryotube, and frozen at -80°C until MDA analysis. After collection of the colon specimens, all animals were subjected to euthanasia with a lethal dose of thiopental.

### Histological analysis

The colon specimens used for histological analysis were kept in neutral formaldehyde for 24 h. Then, they were dehydrated by exposure to ethanol, xylene, for later making the paraffin blocks. Three days later, sections of tissue cut at 5-μm thickness were mounted on a glass slide and stained with hematoxylin-eosin (HE). The HE staining aimed to evaluate the presence and intensity of colitis. A pathologist who did not know the objectives of the study analyzed all slides with an optical microscope at magnification of x200.

The inflammatory score was evaluated using a histological injury scale, which considered three histological parameters: diminution of the crypt length, infiltration of the mucosa by neutrophils, and epithelial damage^27^. Each variable was stratified as follows:

- 0: absent, or no alterations;
- +: mild;
- ++: moderate;
- +++: intense.

The final value of all considered variables for each animal was the mean value after analysis of three different optical fields. The sum of the mean values found for the three variables was used to rank the intensity of the inflammatory score for each animal.

### Immunohistochemical staining

To perform immunohistochemical analysis, we used a previously described technique with slight adaptations^28^.

To identify the presence of E-cadherin and β-catenin in previously prepared blocks, sections cut with 5-μm thickness were made from samples collected from rats of the different experimental groups over the two proposed treatment periods (two or four weeks). The sections were arranged on frosted glass slides that had been signalized and labeled according to the experimental group from which the fragment had been removed and the animal number. Then, the slides were submerged in a 1:100 dilution Trilogy (Trilogy™, Cell-Marque™, Sigma-Aldrich, Darmstadt, Germany) solution, at 95°C, for 45 min, to promote deparaffinization, rehydration, and unmasking. At the end of this period, the slides were transferred to a second glass container with the Trilogy solution at the same temperature, and then they were incubated for 10 min.

Subsequently, the Trilogy solution was replaced with distilled water and kept at room temperature for 30 min. The blocking of endogenous peroxidases was performed by incubating the slides in a 3% hydrogen peroxide for 10 min. Then, the blocking of endogenous proteins was promoted by immersion of the sections in a skim milk solution (Molico™, Nestlé, Vevey, Vaud, Switzerland) for 30 min. After, it was rewashed with water and PBS twice for 2 min each.

A primary anti-E-cadherin antibody (Agilent-Dako, Santa Clara, United States; Clone: NCH-38) was used at a 1:100 dilution to react with E-cadherin protein. To identify β-catenin protein, an anti-β-catenin antibody (Agilent-Dako, Santa Clara, United States; monoclonal mouse anti-human β-catenin, Clone β-catenin-1) was used at a 1:200 dilution. The primary antibody of each protein was added to the slides, which were then incubated in a humid chamber for 1 h at ambient temperature. Subsequently, an avidin-biotin system (secondary antibodies) from an LSAB2 + System-HRP Kit (Agilent-Dako, Santa Clara, United States) was used, and there was incubation with each reagent for 35 min.

The sections were washed with PBS twice and visualized with a diaminobenzidine (DAB) + substrate liquid kit (Sigma-Aldrich, Darmstadt, Germany), which involved the dilution of one drop of chromogen in 1 mL of buffer solution. Then, 100 μL of chromogen was added to the sections for an incubation period of 5 min at room temperature. After, the slices were washed in water, exposed to Harris hematoxylin for 30 s, and rewashed until the excess of hematoxylin was entirely removed. Completed this step, they were dehydrated with three absolute alcohol washes, one xylene/alcohol wash, and two xylene washes before being assembled with coverslips and resin.

### Protein tissue quantification

 The tissue content of both proteins identified by immunohistochemistry staining was quantified by computer-assisted image analysis software (NIS-Elements™, Nikon Inc., Tokyo, Japan), on a focal field in which there were at least three complete and contiguous crypts. The software was installed on a computer system with a great image processing capacity. It can identify areas in which the immunostaining was present transforming the color of the immunostaining (brown) into a white color while coloring the remainder of the field in black, creating a binary image. The software then calculated the percentage of white color that was present relative to the black background (pixels/optical field). Thus, the percentage of white color represented the amount of E-cadherin or β-catenin present in each field of view.

The content of both proteins measure in three different fields of segments not exposed to fecal stream was averaged to determine the mean value of each protein level for each animal. The final value was expressed as a percentage by field (%/f).

### Malondialdehyde assay

 The intensity of oxidative tissue damage (OTD) was quantified by measuring thiobarbituric acid reactive substances (TBARs)^17^. Briefly, 1 g of colonic tissue was put in 5 mL of PBS and homogenized by vortexing and ultrasound sonication for 30 s. The process was repeated three times.

After homogenization, 250 μL of the supernatant obtained was transferred to a plastic tube containing 25 μL of 4% methanolic butylated hydroxytoluene. The sample was then mixed with 1 mL of 12% trichloroacetic acid, 1 mL of 0.73% thiobarbituric acid and 750 μL of Tris/HCl buffer. Then, it was incubated in a water bath at 100°C for 60 min.

Concluded this step, the tubes were placed in a glass vat with ice to block the reaction, and 1.5 mL of n-butanol was added. Afterwards, the mixture was again homogenized for 30 s. The samples were separated by centrifugation for 10 min at 5,000 rotations per minute. The supernatant was removed, and the absorbance of the organic phase at 532 nm was analyzed using a spectrophotometer (UV/vis 6105, Jenway, Bibby Scientific, Cheshire, United Kingdom).

### Statistical analysis

 The results for the degree of inflammation were described according to median values. For tissue levels of E-cadherin and β-catenin, as well as the MDA experiments, the results were described as a mean ± standard error. Mann-Whitney test was used to compare the results found among experimental groups. It was established for all tests level of significance of 5% (*p* < 0.05). It was used * to identify *p* values < 0.05 and ** for *p* values < 0.01.

## Results

**Figure 1:**
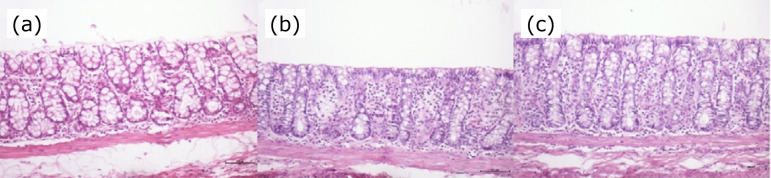
**(a)** Colonic mucosa without fecal stream subjected to treatment with saline for four weeks. **(b)** Colonic mucosa without fecal stream subjected to treatment with SCF (1 g/kg/day) for four weeks. **(c)** Colonic mucosa without fecal stream subjected to treatment with SCF (2 g/kg/day) for four weeks.

Figure 1a shows a sample of colonic epithelium segmented without fecal transit that was subjected to treatment with saline for four weeks. Figure 1b shows a sample of colonic epithelium segmented without fecal stream that was subjected to irrigation with SCF at concentration of 1 g/kg/day, while Figure 1c presents a sample subjected to irrigation with SCF at a concentration of 2 g/kg/day. It was possible to observe the damage of the epithelial surface with ulceration and sinuosity of colonic crypts (HE – x100) in the saline group, as compared to a SCF group, in which the epithelium surface is preserved and exhibits no signs of ulceration and gland alignment (HE – x100).

SCF: sucralfate.

**Figure 2:**
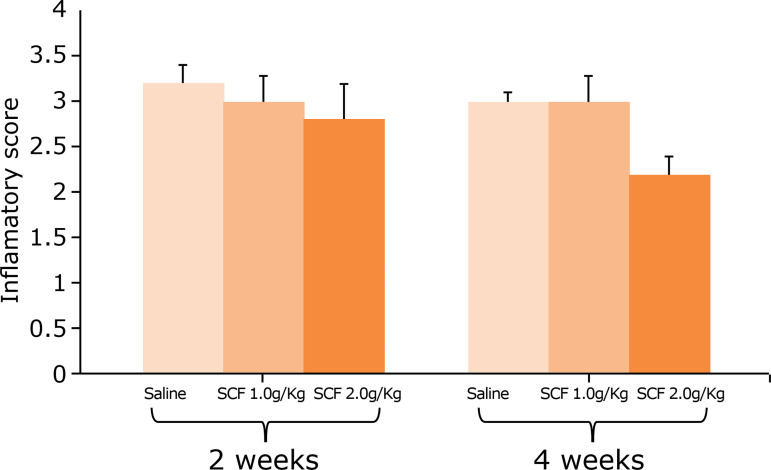
The inflammatory score of animals subjected to daily irrigation with saline, SCF 1 g/kg day and SCF 2 g/kg/day after two and four weeks of treatment. Mann-Whitney test.

Figure 2 shows the inflammatory score of the colonic mucosa without intestinal transit from animals subjected to intervention with saline, SCF 1 g/kg/day andSCF 2 g/kg/day for two and four weeks. As observed, SCF enemas for four weeks were associated with a significant reduction in the inflammatory score as compared to saline enemas.

SCF: sucralfate; *p < 0.05 (SCF 2 g/kg/day < saline).

**Figure 3:**
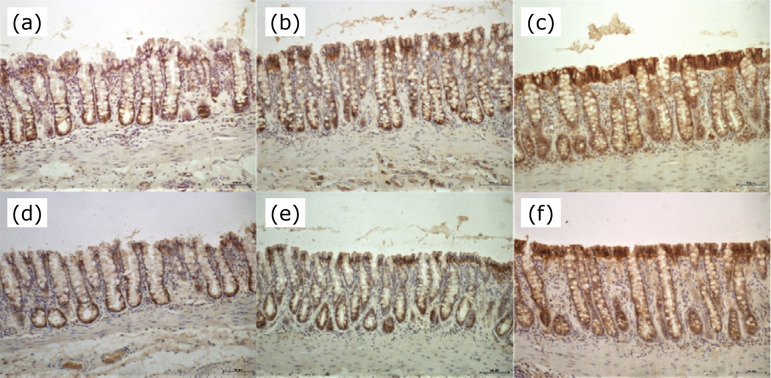
**(a)** Immunostaining of E-cadherin in colonic epithelium without fecal stream subjected to treatment with saline for four weeks. **(b)** Immunostaining of E-cadherin in colonic epithelium without fecal stream subjected to treatment with SCF at concentration of 1 g/kg/day for four weeks. **(c)** Immunostaining of E-cadherin in colonic epithelium without fecal stream subjected to treatment with SCF at concentration of 1 g/kg/dayfor four weeks. **(d)** Immunostaining of β-catenin in colonic epithelium devoid of the fecal stream subjected to intervention with saline for four weeks. **(e)** Immunostaining of β-catenin in colonic mucosa without fecal stream subjected to treatment with SCF at concentration of 1 g/kg/day for four weeks. **(f)** Immunostaining of β-catenin in colonic mucosa without fecal stream exposed to SCF at concentration of 2 g/kg/day for four weeks (IH – x100).

Figure 3a shows the pattern of tissue expression of E-cadherin in the colonic epithelium excluded from the fecal stream, subjected to treatment with saline. Figures 3b and 3c show the pattern of tissue expression of E-cadherin in the colonic epithelium excluded from the fecal stream in those animals submitted to intervention with SCF at concentration of 1 or 2 g/kg/day for four weeks. It was possible to observe in animals subjected to treatment with saline (Fig. 3a) that there was reduction in the expression of E-cadherin on the epithelial surface.

SCF: sucralfate.

In a different way, in animals submitted to intervention with SCF (Fig. 3 b-c), the immunostaining of E-cadherin was maintained, mainly in the apical surface of the colonic glands. Figure 3d shows the pattern of tissue expression of β-catenin in the colonic mucosa devoid from the fecal stream, subjected to treatment with saline. Figures 3e and 3f show the pattern of tissue expression of β-catenin in the colonic mucosa excluded from the fecal stream in those animals submitted to intervention with SCF at concentration of 1 or 2 g/kg/dayfor four weeks. It was possible to observe in animals subjected to treatment with saline (Fig. 3d) reduction in the expression of β-catenin on the apical of the colonic epithelium. However, in animals submitted to intervention with SCF (Fig. 3 b-c), the immunostaining of β-catenin was maintained, mainly in the apical surface of the colonic glands.

Table 1 shows the mean values, with respective standard errors, of the tissue contents of E-cadherin and β-catenin and the levels of MDA in the colonic mucosa without fecal transit in animals subjected to intervention with saline, SCF 1 g/kg/day or SCF 2 g/kg/day for two or four weeks. Tissue levels of both proteins in animals subjected to treatment with SCF, regardless the concentration utilized, was higher than in animals subjected to treatment with saline for two and four weeks. Animals subjected to treatment with saline had higher tissue levels of MDA as compared to those subjected to intervention with SCF, no matter neither the doses used nor the time of treatment.

**Table 1:** Tissue content of E-cadherin, β-catenin and MDA in colonic mucosa devoid of fecal stream submitted to intervention with daily enemas with saline or SCF at concentration of 1 or 2 g/kg for two or four weeks.

	2 weeks				4 weeks		
	Saline	SCF 1 g	SCF 2 g		Saline	SCF 1 g	SCF 2. g
	(M ± SE)						
E-cadherin (%/field)	3.80±0.43	6.61±1.20 * a	8.76±1.16 ** b		4..5±0.20	7.38±0.64 ** c	7.82±0.80 ** d
β-catenin (%/field)	3.81±0.38	6.12±0.50 ** e	7.52±0.44 ** f		4.71±0.22	6.84±0.64 * g	8.63±0.70 * h
MDA × μg × 10^2^	5.59±0.34 ** j	1.74±0.10	2.03±0.17		7.22±0.63 ** k	1.52±0.11	1.99±0.08

MDA: malondialdehyde; SCF: sucralfate; M: mean; SE: standard error;

* p < 0.05 (control vs. SCF);

** p < 0.01 (control vs. SCF);

a p = 0.04;

b p = 0.002;

c p = 0.002;

d p = 0.01;

e p = 0.001;

f p = 0.0001;

g p = 0.04;

h p = 0.02;

^I^p = 0.002;

j p = 0.002;

k p = 0.0001;

Mann-Whitney test.

## Discussion

Studies using experimental models of DC showed that overproduction of ROS by colonic epithelium cells without regular supply of SCFAs could cause a breakdown of the defense mechanisms in the epithelial barrier^5^^,^ 30. Damage to these defense systems allows the migration of luminal harmful pathogens to the sterile internal layers of the colonic wall, triggering and perpetuating the inflammatory process in the colonic mucosa that characterizes DC^5^. To oppose antigen and bacterial infiltration, leucocytes migrate to the site, further increasing ROS formation and worsening mucosal inflammation^2^.

Initially, it was demonstrated that oxidative damage can modify the expression and tissue levels of mucins, which are the primary proteins present in the mucus layer of the colonic mucosa^11^^,^ 28. These studies with models of DC also showed that overproduction of ROS, leading to an oxidative stress, can damage proteins that form the intercellular junction systems. It has already been demonstrated that colonic segments without fecal transit have significant reduction in the tissue content of the main protein constituents of the AJs^13^. Reduction in the levels of these proteins was related to worsen the inflammation of the colonic mucosa and to increase levels of oxidative stress^13^.

Oxidative damage due to overproduction of ROS is considered one of the principal molecular mechanisms involved in the breakdown of the defense system of the epithelial barrier^5^. Thus, it is correct to suppose that the application of clysters containing substances with antioxidant propriety to address the colonic segments devoid of the intestinal transit could minimize deleterious effects of ROS^5^^,^ 11 ^,^ 30.

To evaluate this possibility, studies with experimental models of DC showed that the use of enemas with mesalazine, and n-acetylcysteine, which is a substance with recognized antioxidant activity, reduced not only the inflammatory process in segments without fecal stream, but also the levels of oxidative stress, improving the inflammation of the colonic mucosa^30^^,^ 31. Despite the beneficial effects of these antioxidant drugs in preserving the systems of defense in the colonic epithelial barrier and reducing the levels of oxidative stress, the low ability of these substances to adhere to the epithelial surface meant that they had to be applied several times a day^27^^,^ 29.

The application of enemas several times a day could limit the acceptance of the use of these substances in clinical practice. The possibility of finding a substance with antioxidant and anti-inflammatory action, as well as the high capacity to adhere firmly to the damaged colonic epithelial surface, could overcome this obstacle.

SCF is employed in the treatment of different gastrointestinal diseases to reduce inflammation of the epithelial surface and favor the healing process^18^. SCF join tightly to the inflamed mucosa, forming a stable and insoluble layer that protect damaged epithelium^20^^,^ 21. SCF improves the healing of inflamed mucosa, has an antioxidant and anti-inflammatory properties, and stimulates the production of EGF^18^^-^ 24. Authors who evaluated the effects of SCF using models of chemically-induced colitis have shown that the application of clysters with SCF increases mucosal repair, probably through increasing mucus production, which favors epithelial healing^31^^,^ 32. Due to these properties, the use of enemas with SCF has been considered a promising substance for the treatment of other forms of colitis^24^^,^ 25 ^,^ 33. Clinical studies confirm the benefits of enemas containing SCF for the treatment of radiation proctitis and ulcerative colitis^18^^-^ 25 ^,^ 31 ^-^ 34 ^,^.

Recently, other authors have evaluated the effects of enemas with SCF to treat the experimental DC^14^^,^ 27 ^-^ 29. These studies showed that SCF reduces the inflammatory process, decreases neutrophil infiltration and oxidative tissue damage in colonic segments devoid of exposure to the fecal stream^29^. In addition to the capacity of SCF to stimulate the production of mucins and EGF, it was demonstrated that the use of SCF enemas stimulates the production of different subtypes of colonic mucins by cells of the colonic epithelium, favoring mucosal healing^14^^,^ 27 ^-^ 29.

These results confirm the effectiveness of SCF in stimulating the production of the mucins and in reducing the oxidative damage and the inflammation of the intestinal mucosa^14^^,^ 26 ^-^ 28. Despite the beneficial effects of SCF enemas for reducing inflammation and oxidative stress of the colonic mucosa without intestinal transit, no study has evaluated the effects of the SCF in preserving the proteins that form the AJs. If SCF is able to preserve the integrity and the content of these proteins, it would be additional favorable evidence for the indication of enemas with SCF in the treatment of patients with DC.

The results found in this study reinforce the beneficial effects of the use of clysters with SCF for the treatment of experimental DC. We demonstrated that the animals subjected to enemas with SCF presented an increase in mucosal levels of E-cadherin and β-catenin compared with levels in animals subjected to treatment with saline. The increase in both protein levels was independent of the concentration used and the treatment time adopted. An increase in the levels of both proteins was related to improvement in the degree of mucosal inflammation and decrease in neutrophil infiltration and in the tissue levels of MDA. A previous study showed an increase in the tissue levels of MDA in colonic mucosa without fecal transit, which increased the extent of colonic exclusion^35^.

The results of this study showed that, regardless the SCF concentration used and the proposed treatment time, the tissue levels of MDA were always lower than they were in animals that received enemas with saline. These findings seem to confirm the results of previous studies, which showed the critical antioxidant activity of the SCF^18^^,^ 35. The reduction in oxidative stress is related to the improvement in the grade of mucosal inflammation and the increase in the tissue content of E-cadherin and β-catenin. It has already been demonstrated that E-cadherin levels are significantly reduced in colonic mucosa without fecal stream, and this reduction is related to the time of colonic exclusion, levels of oxidative tissue damage, and the severity of mucosal inflammation^13^.

It is important to highlight that, although computer-assisted image analysis (computerized morphometry) is a widely used methodology in measuring tissue content of proteins identified by immunohistochemistry, other techniques could be employed, such as western-blotting or enzyme-linked immunosorbent assay. However, these methods do not allow identification of the correct site of expression of the proteins in tissue. These techniques only identify the total protein content in a certain amount of tissue. For this reason, we preferred to use immunohistochemistry to accurately identify the site of expression of the proteins and the computer-assisted image analysis to determine their tissue content. Other authors have also used the same methodology to identify other proteins^13^^,^ 14 ^,^ 27 ^,^ 28.

This study is associated to some limitations, which need to be emphasized in the results’ analysis. Our study was carried out in an experimental model in rats. Even though most experimental studies regarding colitis models use rats as experimental animals, it is difficult to ensure that the same results can be replicated in humans. Apart from that, the findings of this study confirm that the use of clysters with SCF was able to preserve the tissue contents of the main proteins that form AJs, and decrease the oxidative damage and inflammatory process of the colonic mucosa in rats devoid of intestinal transit.

The results of this study suggest the beneficial effects of the use of SCF in experimental DC. Despite that, clinical studies are still necessary to verify if enemas using SCF is a beneficial therapeutic strategy for the treatment of the disease in humans.

## Conclusion

SCF reduces the inflammation and oxidative stress and increases the tissue content of E-cadherin and β-catenin in colonic mucosa devoid to the fecal stream.
